# Gut Microbe-Generated Metabolite Trimethylamine-N-Oxide and Ischemic Stroke

**DOI:** 10.3390/biom14111463

**Published:** 2024-11-18

**Authors:** Zhen Li, Xinyi He, Qi Fang, Xulong Yin

**Affiliations:** Department of Neurology, The First Affiliated Hospital of Soochow University, No. 899 Pinghai Road, Suzhou 215006, China; 20237832009@stu.suda.edu.cn (Z.L.); 20235232075@stu.suda.edu.cn (X.H.)

**Keywords:** trimethylamine-N-oxide, ischemic stroke, gut microbiota, mechanisms, interventions

## Abstract

Trimethylamine-N-oxide (TMAO) is a gut microbiota-derived metabolite, the production of which in vivo is mainly regulated by dietary choices, gut microbiota, and the hepatic enzyme flavin monooxygenase (FMO), while its elimination occurs via the kidneys. The TMAO level is positively correlated with the risk of developing cardiovascular diseases. Recent studies have found that TMAO plays an important role in the development of ischemic stroke. In this review, we describe the relationship between TMAO and ischemic stroke risk factors (hypertension, diabetes, atrial fibrillation, atherosclerosis, thrombosis, etc.), disease risk, severity, prognostic outcomes, and recurrence and discuss the possible mechanisms by which they interact. Importantly, TMAO induces atherosclerosis and thrombosis through lipid metabolism, foam cell formation, endothelial dysfunction (via inflammation, oxidative stress, and pyroptosis), enhanced platelet hyper-reactivity, and the upregulation and activation of vascular endothelial tissue factors. Although the pathogenic mechanisms underlying TMAO’s aggravation of disease severity and its effects on post-stroke neurological recovery and recurrence risk remain unclear, they may involve inflammation, astrocyte function, and pro-inflammatory monocytes. In addition, this paper provides a summary and evaluation of relevant preclinical and clinical studies on interventions regarding the gut-microbiota-dependent TMAO level to provide evidence for the prevention and treatment of ischemic stroke through the gut microbe–TMAO pathway.

## 1. Introduction

Strokes are cerebrovascular accidents characterized by high incidence, disability, and mortality rates. They represent the second leading cause of death worldwide, with nearly 87% being ischemic [1,2]. Ischemic stroke is caused by acute cerebral blood circulating disorders that result in local ischemia and hypoxia in brain tissue, forming limited necrosis or softening. Ischemic stroke poses a significant health threat and financial burden [3,4]. Although great progress has been made in the prevention, treatment, and rehabilitation of this condition, many challenges remain [5,6,7,8]. First, traditional risk factors have a limited ability to predict disease and do not provide adequate warning. Second, the current effectiveness of revascularization therapy is limited by its short time window and increased risk of bleeding and ischemia–reperfusion injury. Third, due to the lack of clarity around the mechanism responsible for ischemic stroke, specific drugs to inhibit the progression of the disease and promote the recovery of neuronal function are lacking [9]. Therefore, it is necessary to identify new risk factors and explore the pathophysiological mechanisms related to ischemic stroke to reduce the incidence and improve the prognosis associated with this condition.

A diverse community of micro-organisms lives in the human gut and plays important roles in the maintenance of normal physiological functions, homeostasis of the internal environment, and the occurrence and development of diseases. Numerous studies have demonstrated bidirectional interactions between the brain and gut microbiota [10,11,12]. Recent studies have found that gut microbiota and their metabolites regulate the progression of many neurological disorders, including strokes [13,14,15]. Disorders of the gut microbiota and their metabolites change the intestinal environment and affect nutrient absorption, substance metabolism, and immune balance, thereby increasing the risk (through hypertension, diabetes, etc.) of developing ischemic stroke in different ways [16,17,18]. In addition, these disorders affect the severity and prognosis of ischemic stroke by regulating immunity, promoting infection and inflammation, and promoting thrombosis [13,19,20]. Ischemic stroke also affects the internal balance of the gut microbiota. The genomic composition of the gut microbiota in ischemic stroke patients differs from that of healthy people [20,21,22], suggesting that homeostasis of the microbiota is disrupted by ischemic stroke, resulting in a switch in the secretion of metabolites toward noxious ones.

**Figure 1:**
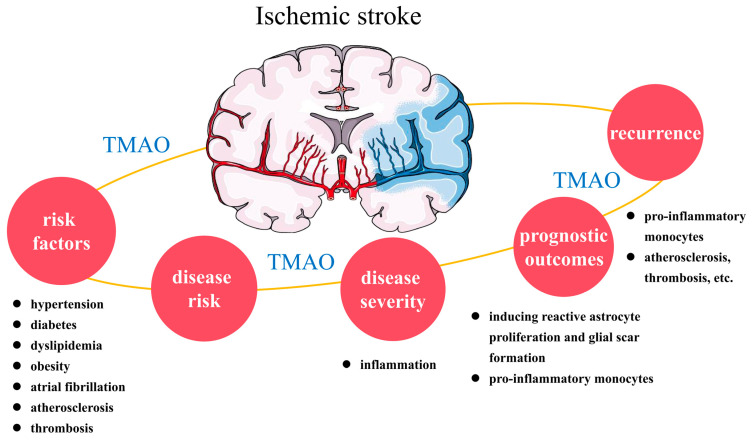
The relationship between TMAO and ischemic stroke. TMAO is associated with ischemic stroke risk factors (hypertension, diabetes, dyslipidemia, obesity, atrial fibrillation, atherosclerosis, and thrombosis), disease risk, severity, prognostic outcomes, and recurrence. The pathogenic mechanisms underlying the aggravation of the disease severity by TMAO and its effects on prognostic outcomes and recurrence risk remain unclear but may involve inflammation, astrocyte function, and pro-inflammatory monocytes. TMAO: trimethylamine-N-oxide.

A variety of microbiota-derived metabolites (noradrenaline, tryptophan, γ-aminobutyric acid, trimethylamine-N-oxide (TMAO), short-chain fatty acids, etc.) can act directly on the central nervous system (CNS) or communicate with the CNS indirectly via the vagus nerve, spinal cord, endocrine and immune systems, and other pathways. Importantly, gut-microbiota-derived TMAO in the peripheral circulation can cross the blood–brain barrier, directly activate nerve cells, and modulate neuroinflammation, thus playing roles in a variety of neurological disease mechanisms [23,24]. Among the gut microbial metabolites, TMAO, short-chain fatty acids, and bile acids are involved in stoke occurrence and development [19,22]. This article focuses on the relationship between TMAO and ischemic stroke. We review the role of TMAO in disease-related risk factors, disease risk, severity, prognosis, and recurrence (Figure 1) and evaluate intervention strategies, aiming to provide evidence and insights to improve the management of ischemic stroke.

## 2. Generation and Metabolism of TMAO

Trimethylamine-N-oxide is a small organic compound that is soluble in water and blood. Its biosynthesis in humans depends on three main factors: dietary intake, gut microbial metabolism, and endogenous processing in the liver. Free TMAO in seafood can be absorbed directly into the bloodstream via the gastrointestinal tract, without metabolism [25]. Trimethylamine (TMA), a precursor of TMAO, is synthesized in vivo from compounds containing TMA groups such as phosphatidylcholine, betaine, and carnitine, which are found in foods such as red meat, poultry, liver, eggs, and dairy products. In addition, the above metabolic processes are regulated by gut microbial enzymes. Furthermore, TMA absorbed from the gut into the portal vein is oxidized to TMAO via flavin monooxygenase (FMO)1 and FMO3 in the liver. The diet, gut microbiome, and liver together comprise the biosynthetic pathway for TMAO. Therefore, dietary choices, the abundance and activity of gut microbiota, and FMO are key factors affecting TMAO synthesis in vivo. Accordingly, vegetarians with low plasma TMAO levels have a lower risk of developing cardiovascular and cerebrovascular diseases than omnivorous humans [26,27]. Multiple gut bacteria from the phyla *Firmicutes* and *Proteobacteria* are involved in TMAO production. Gut bacteria that utilize key enzymes (choline utilization TMA-lyase system (cutC/D), the carnitine Rieske-type oxygenase/reductase system (cntA/B), Rieske-type oxygenase/reductase (yeaW/X), etc.) to convert carnitine, betaine, and choline into TMA are important, including *Lachnoclostridium*, *Clostridium*, and *Escherichia* [28,29]. In addition, the expression of FOM3, a key enzyme responsible for 90% of TMA oxidation, is influenced by sex but not ethnicity [30]. Women with high FMO3 expression and high plasma TMAO levels are more prone to the development of atherosclerosis than men. Interestingly, although previous reports suggested that almost all TMA is oxidized to TMAO in the liver, recent findings by Koay et al. suggest that, in addition to oxidation by hepatic FMO, TMAO is also generated by direct oxidation in the gut. The gut itself is another important site of TMAO oxidative production. The TMAO concentration in feces is similar to plasma concentrations in mice, and a decrease in FMO3 expression is accompanied by an increase in plasma TMAO after a chronic high-choline diet. In addition, plasma TMAO concentrations have been positively correlated with abundant *Enterorhabdus*. This genus can degrade mucin, leading to direct contact between commensal bacteria and the gut epithelium and ultimately increasing antimicrobial reactive oxygen species (hydrogen peroxide, superoxide, etc.) generation in the gut, leading to the oxidation of TMA to TMAO [31].

More than 95% of TMAO is cleared by the kidneys in its prototype form [32,33]. Therefore, the excretory function of the kidneys seriously affects the plasma TMAO level. Interestingly, chronic dietary red meat consumption can reduce renal TMAO excretion, ultimately increasing the systemic TMAO level [34].

## 3. TMAO and Ischemic Stroke Risk Factors

**Figure 2:**
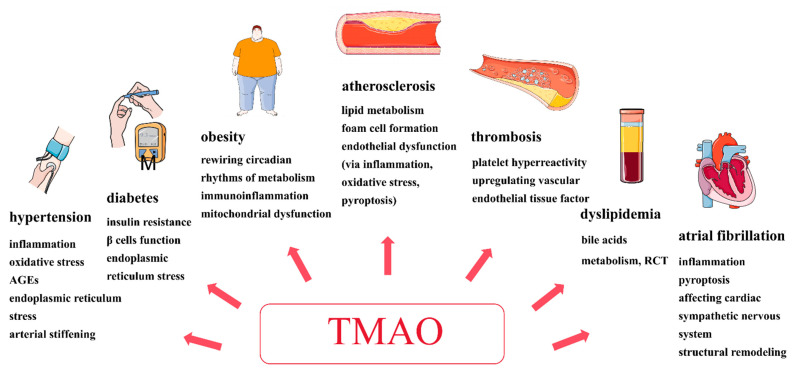
TMAO plays an important role in ischemic stroke risk. It may be involved in the pathogenesis of diseases such as hypertension, diabetes, dyslipidemia, obesity, atrial fibrillation, atherosclerosis, and thrombosis, ultimately increasing the ischemic stroke risk. Importantly, TMAO induces atherosclerosis and thrombosis through several mechanisms, including lipid metabolism, foam cell formation, endothelial dysfunction (via inflammation, oxidative stress, pyroptosis), enhanced platelet hyper-reactivity, and the upregulation and activation of vascular endothelial tissue factor. AGEs: advanced glycation end products, RCT: the reverse cholesterol transport, TMAO: trimethylamine-N-oxide.

Multiple diseases and unhealthy lifestyle habits increase ischemic stroke risk. There is increasing evidence that disturbances of the gut microbiota and its derived metabolites are associated with ischemic stroke risk. Recent studies have found that TMAO may be involved in the pathogenesis of diseases such as hypertension, diabetes, dyslipidemia, obesity, atrial fibrillation, atherosclerosis, and thrombosis (Figure 2), ultimately increasing the ischemic stroke risk.

### 3.1. Hypertension

Hypertension is an important risk factor associated with ischemic cerebral infarction, as it promotes atherosclerosis, thrombosis, vasospasm, hemodynamic changes, microvascular dysfunction, and other pathologic pathways. A meta-analysis including 6176 hypertensive individuals showed that circulating TMAO levels were dose-dependently and positively related to the hypertension risk, and the relative risk (RR) for its prevalence increased by 9% per 5 μmol/L increase in the TMAO concentration [35]. TMAO treatment increases the release of intracellular Ca^2+^ induced by angiotensin II in afferent arteries and vascular smooth muscle cells via the PERK/ROS/CaMKII/plc-β3 axis, thereby promoting angiotensin II-induced vasoconstriction and hypertension [36]. In addition, long-term high-salt diets are known to promote the occurrence of hypertensive diseases. Liu et al. showed that TMAO elevation caused by excessive dietary salt intake in the bloodstream and central nervous system increases oxidative stress and inflammation in the hypothalamic paraventricular nucleus, facilitating sympathetic excitation and hypertension development [37]. Middle-aged and elderly people showed higher plasma TMAO levels, which were independently and positively correlated with the carotid–femoral pulse wave velocity (a measure of arterial stiffness) and systolic blood pressure (SBP). TMAO supplementation increased elastic artery stiffening and SBP by increasing the abundance of advanced glycation end products (AGEs) in mice [38]. Hypertension in offspring, programmed by maternal chronic kidney disease, was associated with increases in plasma TMAO and gut microbiota disorders. These could be prevented by the TMAO inhibitor iodomethylcholine (IMC) [39]. Furthermore, TMAO may be involved in the progression of hypertension-related complications. The study found higher TMAO concentrations and more severe white matter lesions in rats with spontaneous hypertension than in normal controls. TMAO promoted brain white matter demyelination by increasing oligodendrocyte pyroptosis [40]. However, Tomasz et al. found that low-dose supplementation with TMAO as a protective penetrant reduced diastolic dysfunction and cardiac fibrosis in hypertensive rats, indicating the beneficial effects of low-dose TMAO treatment on the cardiovascular system [41]. In conclusion, there is no sufficient evidence to show that TMAO has a direct effect on hypertension, but it does influence the pathogenesis and promote the occurrence and progression of the disease, as well as being associated with disease complications.

### 3.2. Diabetes

TMAO plays an important role in the onset and progression of diabetes, an important risk factor for ischemic stroke that affects the disease prognosis. Diabetes increases the ischemic stroke risk through multiple mechanisms, including the promotion of atherosclerosis, endothelial dysfunction, insulin resistance, and coagulation system dysfunction. A cohort study from China with a median follow-up period of 8.9 years associated higher serum TMAO concentrations with elevated fasting blood glucose and type 2 diabetes (T2D) [42]. The correlation remained even after adjustment for diet and physical activity [43]. On the other hand, previous studies on the relationship between TMAO and gestational diabetes mellitus (GDM) showed inconsistent results. Plasma TMAO levels were positively associated with GDM among participants at 24–32 weeks of gestation [44]. However, another study identified a negative association between these two factors when TMAO ≤ 16 nmol/mL [45]. TMAO may be involved in diabetes-related mechanisms by influencing insulin resistance, β-cell function, and inflammation. It has also been associated with insulin resistance in mouse models and clinical patients [46,47]. In overweight individuals who participated in a dietary intervention, reduced circulating TMAO levels were associated with improved insulin sensitivity [48]. Chen et al. found that TMAO induced the expression of the transcription factor fox01 by binding to and activating PERK (endoplasmic reticulum stress kinase), ultimately driving the development of diabetes [49]. TMAO was shown to decrease the β-cell proportion, reduce glucose-stimulated insulin secretion, and impair glucose tolerance by promoting β-cell apoptosis, inhibiting calcium transients, and inducing Serca2 loss [50]. However, Emily et al. suggested that TMAO protects β-cell function by inhibiting T2D-like glucolipotoxic-mediated damage, which is beneficial for individuals with diet-induced type 2 diabetes [51]. Previous contradictory findings warrant a further exploration of TMAO’s effects on β cells. TMAO increases the risk of diabetes complications and worsens the prognosis. Higher TMAO concentrations have been associated with major adverse cardiovascular events, including stroke [52], as well as glycemic variability in diabetic patients with cerebral infarction [53]. TMAO also increases the risk of diabetic retinopathy and is related to disease severity [54]. It is not only associated with diabetic kidney disease (DKD) but also increases the risk of all-cause mortality in patients with DKD [55,56,57]. In patients with type 2 diabetes, elevated serum TMAO levels have been associated with non-alcoholic steatohepatitis [58]. TMAO also affects the prognosis of patients with type 1 diabetes, of which the pathogenesis is different from that present in type 2 diabetes. A study including 1159 patients with type 1 diabetes found that after adjustment for traditional cardiovascular risk factors, higher plasma TMAO levels were related to all-cause and cardiovascular mortality, cardiovascular diseases, and end-stage renal disease [59].

Overall, although some findings are contradictory, there is increasing evidence that TMAO influences the onset and maintenance of diabetes through a variety of pathophysiological mechanisms and promotes the occurrence of many complications, including cardiovascular diseases, stroke, diabetic retinopathy, diabetic kidney disease, and non-alcoholic steatohepatitis, ultimately increasing the risk of mortality.

### 3.3. Dyslipidemia

TMAO is closely related to dyslipidemia, a condition that increases the ischemic stroke risk by promoting atherosclerosis, inducing blood vessel narrowing and thrombosis. FMO3 is a key enzyme in TMAO production, and its knockdown not only reduces the circulating TMAO concentration but also leads to changes in lipid and cholesterol metabolism [46,60]. TMAO is not only positively correlated with the serum triglyceride level; it is also negatively correlated with the serum high-density lipoprotein cholesterol (HDL-C) level [61,62]. Both TMAO and lipid levels were shown to decrease after rosuvastatin treatment [61]. However, there appears to be no correlation between TMAO and low-density lipoprotein cholesterol (LDL-C), a key factor in atherosclerosis promotion [63]. TMAO is involved in cholesterol metabolism and affects reverse cholesterol transport (RCT). It inhibits bile acid transport by activating small heterodimer partner (SHP) and nuclear farnesoid X receptor (FXR), ultimately reducing cholesterol absorption and decreasing RCT [64]. Although the results of previous studies are inconsistent [27,65], TMAO seems to affect cholesterol metabolism by reducing the expression of intestinal transporters [27]. In addition, Yang et al. found that TMAO can promote hyperlipidemia acute pancreatitis via the Toll-like receptor (TLR)/p-glycoprotein 65 (p65) signaling pathway [66]. On the other hand, a high-fat diet led to lower circulating TMAO levels via changes in the gut microbiota and FMO3 expression inhibition [67]. Recently, several drugs have been shown to affect cholesterol metabolism and reduce dyslipidemia through the TMAO pathway. Black raspberry extracts alleviated hypercholesterolemia via lowering TMAO levels in vivo [68]. TMAO is also involved in the mechanism by which various traditional Chinese medicines regulate lipid metabolism [69]. It may also play a role in the mechanism by which probiotics alleviate hypercholesterolemia [70]. Although previous findings are not entirely consistent, TMAO is indeed associated with dyslipidemia. Strong evidence that TMAO directly affects dyslipidemia is still needed.

### 3.4. Obesity

Obesity is an important risk factor for cerebral infarction that increases stroke risk. Here, we summarize recent findings on TMAO and obesity. Circulating TMAO levels are higher in obese participants [71], and short-term high-fat diet supplementation has been associated with increased postprandial concentrations of TMAO [72]. The plasma TMAO concentration increases with increases in the body mass index (BMI) [73], visceral adiposity index (VAI), and fatty liver index (FLI) [74]. Additionally, the TMAO concentration is predictive of future obesity [75]. However, there are conflicting results [76]. The pathological mechanism by which TMAO interacts with obesity involves the rewiring of circadian rhythms associated with metabolism, immunoinflammation activation, and mitochondrial dysfunction. Targeting the TMAO inhibitor (cutC inhibitor) may reduce diet-induced obesity by reorganizing host circadian rhythms associated with metabolism [77]. TMAO promotes the differentiation of 3T3-L1 preadipocytes into adipocytes and increases the expression of pro-inflammatory cytokines in macrophages infiltrating adipose tissue [78]. On the other hand, a high-fat diet was shown to enhance the respiration-dependent choline catabolism of E. coli by impairing mitochondria bioenergetics in the intestinal epithelium and enhancing the enteric bioavailability of oxygen and nitrate, ultimately increasing the plasma TMAO concentration [79]. In addition, TMAO was shown to exacerbate obesity-related diseases and was associated with atherosclerosis and arterial stiffness development in children with obesity [71]. TMAO was found to contribute to obesity-related cardiac dysfunction via pro-inflammatory responses and the aggravation of cardiac fibrosis [80]. Finally, a variety of weight loss methods, including plant-based diets, lifestyle interventions, and capsanthin extract treatment, can reduce TMAO levels [81]. In conclusion, TMAO plays a role in obesity development, which affects the risk of ischemic stroke.

### 3.5. Atrial Fibrillation

Atrial fibrillation (AF), one of the most common arrhythmias, increases the ischemic stroke risk by promoting intra-atrial thrombosis and embolus shedding, inducing cerebral embolism. The concentration of bacterial genes involved in the biosynthesis of TMA, a precursor to TMAO, was shown to be greater in the guts of AF patients [82]. Elevated circulating TMAO concentrations were associated with an increased AF risk in patients with suspected stable angina and elderly participants [83]. However, in participants with a high cardiovascular risk, TMAO concentrations were not associated with AF [84]. A bidirectional Mendelian randomization study showed similar results [85]. TMAO exacerbated acute electrical remodeling induced by autonomic remodeling by activating the p65 nuclear factor kappa-B (NF-κB) signaling pathway and increasing the inflammatory cytokine concentration in the atrial ganglionated plexus, ultimately promoting AF progression [86]. Cold exposure increased atrial M1 macrophage infiltration and the expression of Caspase-1-p20 and cleaved-Gasdermin D (a marker of pyroptosis) by changing the gut bacterial composition and increasing the TMAO concentration, ultimately leading to atrial structural remodeling and AF induction [87]. TMAO was shown to exacerbate atrial fibrosis through the Wnt3a/beta-catenin signaling pathway [88]. In addition, TMAO may promote diabetes-related AF by exacerbating atrial inflammation and connexin remodeling [89]. Overall, TMAO may aggravate atrial structure remodeling and electrical remodeling by activating inflammatory signaling pathways, affecting the cardiac sympathetic nervous system, increasing pro-inflammatory cell infiltration, and inducing pyroptosis, ultimately leading to the pathogenesis and maintenance of atrial fibrillation. TMAO also promotes AF complications and worsens the prognosis. Importantly, higher circulating TMAO concentrations have been associated with thrombosis formation in atrial fibrillation patients [90], and this is a key risk factor for cardiogenic stroke. In atrial fibrillation patients, higher TMAO concentrations have been associated with diabetes, heart failure, and larger, more severe cerebral infarcts, as well as increased total mortality and cardiovascular mortality rates [91]. Although the relationship between TMAO and AF has been demonstrated, evidence supporting a direct effect is lacking.

### 3.6. TMAO and Atherosclerosis

Atherosclerosis is closely related to ischemic stroke, and atherosclerosis of the carotid and cerebral arteries increases the cerebral infarction risk. There is mounting evidence to show that TMAO promotes the occurrence and development of atherosclerosis through complex interactions between cholesterol metabolism, foam cell formation, and endothelial dysfunction (via inflammation, oxidative stress, pyroptosis). Preclinical studies have shown that supplementation with a high choline dose or TMAO increases TMA/TMAO production in vivo by changing the gut microbiome composition, ultimately increasing the atherosclerosis risk [27]. Atherosclerosis susceptibility was transmitted via the transplantation of gut microbiota from high-TMAO mice to low-TMAO mice [92]. The above relationships among diet, gut microbiota, and TMAO have also been demonstrated in clinical trials [25]. The altered microbiota associated with diets rich in choline and L-carnitine was associated with elevated circulating TMAO concentrations in humans, which played an important role in the pathogenesis of atherosclerosis and increased the ischemic stroke risk. Next, we focus on the pathogenic mechanisms underlying TMAO-induced atherosclerosis. 

#### 3.6.1. Cholesterol Metabolism

The relationship between TMAO and dyslipidemia has been confirmed by several studies. Dyslipidemia, particularly abnormal cholesterol levels, is a key driver of atherosclerosis. In the theory of atherosclerotic lipid infiltration, elevated circulating cholesterol levels and their infiltration are important initial processes associated with atherosclerotic plaque formation. TMAO aggravates atherosclerotic plaques by reducing reverse cholesterol transport (RCT) [27], the process by which cholesterol is transported from peripheral tissues (such as artery walls) back to the liver to be cleared. Although the mechanism by which TMAO reduces RCT is unclear, it may involve bile acid metabolism and HDL-C function. Bile acid synthesis is one of the main cholesterol excretion pathways. In preclinical studies, mice supplemented with dietary TMAO were found to have downregulated expression of the key bile acid synthetic enzymes (Cyp7a1 and Cyp27a1) and bile acid transporters (Oatp1, Oatp4, Mrp2, and Ntcp) [27]. A recent study found that bile acid synthesis was inhibited by TMAO via activation of small heterodimer partner (SHP) and farnesoid X receptor (FXR) [64]. In mice, TMAO has been negatively related to HDL-C, which mediates RCT [93]. Although the results of previous clinical studies are inconsistent, recent studies have negatively associated TMAO with HDL-C [61,62]. A rosuvastatin intervention was shown to significantly reduce plasma TMAO, triglyceride, and LDL-C levels, but it increased HDL-C levels in patients with atherosclerotic cardiovascular disease. Moreover, patients in the high-TMAO group exhibited a greater reduction in plasma TMAO levels after the rosuvastatin intervention than patients in the low-TMAO group, suggesting that there is an important relationship between TMAO and statins [61]. TMAO may reduce RCT by inhibiting HDL-C function by promoting cholesterol efflux from macrophages. In addition, TMAO reduces intestinal cholesterol absorption by downregulating the expression of intestinal transporters.

#### 3.6.2. Foam Cell Formation

Foam cell formation is an important pathophysiological feature of early atherosclerosis lesions, which aggravates the inflammatory response during atherosclerosis progression. TMAO upregulates the expression of macrophage scavenger receptors (lectin-like oxLDL receptor-1, scavenger receptor A1, CD36), promoting the phagocytosis of oxidized LDL-C, resulting in cholesterol overload and accumulation in macrophages and ultimately leading to the formation of foam cells [94]. Further studies found that TMAO induces macrophage conversion to foam cells via the CD36/mitogen-activated protein kinase (MAPK)/c-Jun N-terminal kinase (JNK) pathway [95]. Recent research found that the mechanisms underlying TMAO-induced atherosclerosis lesions involve alterations in α-2-macroglobulin, apolipoprotein C1, transmembrane protein 106a, and CD36 [96].

#### 3.6.3. Endothelial Dysfunction

Endothelial injury and dysfunction are central links in the initiation and progression of atherosclerosis. Endothelial cell dysfunction can manifest as dependent vasodilation disorder, oxidative stress enhancement, chronic inflammation, and leukocyte adhesion. The pathogenic mechanisms underlying TMAO-induced endothelial dysfunction involve increased inflammation, oxidative stress, and pyroptosis. In vitro experiments have shown that high levels of TMAO lead to a pro-inflammatory response and oxidative stress of endothelial progenitor cultured cells by increasing the production of inflammatory mediators (interleukin 6, tumor necrosis factor-α) and reactive oxygen species (ROS) and decreasing nitric oxide (NO) production [97]. Recent studies have found that TMAO-enhanced inflammation is associated with the inhibition of the adenosine 5’-monophosphate-activated protein kinase (AMPK) and sirtuin 1 (SIRT1) signaling pathways in endothelial and vascular smooth muscle cells [98]. TMAO was shown to promote vascular endothelial cell pyroptosis by activating the succinate dehydrogenase iron–sulfur subunit B (SDHB)/reactive oxygen species (ROS) pathway, ultimately aggravating atherosclerosis [99]. TMAO was also found to upregulate the expression of inflammatory markers through the nuclear factor-κB (NF-κB) signaling pathway after both a chronic choline diet and acute TMAO injection [100]. Activation of the NOD-like receptor family pyrin domain containing 3 (NLRP3) inflammasome induced by TMAO was found to promote chronic vascular inflammation, an important mechanism in the promotion of atherosclerosis. Chen et al. found that elevated TMAO promotes neointimal formation and vascular remodeling by enhancing endoplasmic reticulum (ER) stress, oxidative stress, and the activation of the inflammasome [101]. Recent studies have found that proline/serine-rich coiled-coil protein 1 (PSRC1) may act as an upstream factor that regulates TMAO production and prevents atherosclerosis formation [102]. TMAO has also been shown to induce vasodilation dysfunction by downregulating NO production and reducing its bioavailability. In addition, cerebrovascular disease risk factors, such as hypertension, diabetes, dyslipidemia, and obesity, which are closely associated with TMAO, can induce endothelial dysfunction through endothelial nitric oxide synthase uncoupling, activation of the renin–angiotensin system, aggravation of endothelial cell senescence, endothelial–mesenchymal transition, oxidative stress, and inflammation. In general, the mechanism associated with TMAO-induced endothelial cell dysfunction involves oxidative stress, chronic inflammation, and cell pyroptosis, ultimately promoting the development and progression of atherosclerosis and increasing the ischemic stroke risk.

Despite the promising findings from recent studies, the definite mechanisms of TMAO-related atherosclerosis are not fully understood. In the future, TMAO pathophysiological mechanisms and targeted therapeutic strategies for the prevention of atherosclerosis need to be further explored.

### 3.7. TMAO and Thrombosis

Thrombosis is a crucial step in the development of ischemic stroke, and it has been shown to be associated with TMAO. TMAO not only increases platelet reactivity and aggregation but also upregulates the expression of vascular endothelial tissue factor (TF), which plays a key role in coagulation reaction. Zhu et al. found that, at physiological levels, TMAO stimulation dose-dependently increases platelet reactivity by enhanced intracellular Ca^2+^ release [103]. In clinical studies, a direct pro-thrombotic effect of TMAO, which can be attenuated by low-dose aspirin, has been demonstrated [104]. TMAO-induced platelet reactivity has been shown to be an independent risk factor for cardiovascular and all-cause mortality in clinical studies [105]. Clopidogrel is widely used in clinical practice to inhibit platelet aggregation and can effectively prevent thrombosis. Recent research found that TMAO increased clopidogrel resistance [106] by reducing the formation of active metabolites and impairing the platelet response to clopidogrel by activating the nicotinamide adenine dinucleotide phosphate oxidase (NOX)-dependent ROS/nuclear factor erythroid 2-related factor 2 (Nrf2)/carboxylesterase 1 (CES1) pathway [107]. The in vivo and in vitro expression of vascular endothelial TF and vascular cell adhesion molecule (VCAM) 1 is enhanced by TMAO [108]. TMAO upregulates vascular endothelial TF expression via the NF-κB signaling pathway and increases TF activity and thrombin production, promoting arterial thrombosis [109].

## 4. TMAO and the Risk of Ischemic Stroke in Clinical Studies

There is mounting evidence from clinical studies to show that high circulating TMAO levels are positively associated with the ischemic stroke risk. Many studies indicate that patients with ischemic stroke have higher circulating TMAO concentrations than controls [110,111,112,113]. A case–control study including 953 patients experiencing their first ischemic stroke found that after adjusting for variables such as age, sex, BMI, hypertension, and diabetes, higher TAMO levels were associated with an increased ischemic stroke risk [110]. After adjusting for variables such as vascular risk factors, disease features, and pre-stroke treatment, the severe ischemic stroke risk (National Institutes of Health Stroke Scale (NIHSS) ≥ 6) was increased by 22%, when the TMAO level was increased by 1 μM [111]. However, plasma TMAO concentrations were unchanged or even lower in large-artery atherosclerotic stroke or transient ischemic attack patients [114,115]. The conflicting results may be attributed to inconsistencies in the study time point, means of treatment, ethnicity, screening for confounding variables, sample size, and constituent ratios of stroke subtypes. Because plasma TMAO levels increase in the acute phase of ischemic stroke and decrease during the first few days [116], it is recommended that TMAO levels are measured during the acute phase of ischemic stroke (<24 h). On the other hand, although the small sample study found no significant difference in plasma TMAO levels among different ischemic stroke subtypes (TOAST criteria), the relationship between plasma TMAO levels and stroke subtypes with different risk factors and pathophysiological mechanisms needs to be further studied. Several cohort studies have found a positive association between elevated TMAO levels and the ischemic stroke risk. A large-sample study with a 15-year median follow-up period showed that, after adjusting for confounding factors, elevated TMAO significantly increased the ischemic stroke risk (hazard ratio (HR) comparing a doubling of TMAO: 1.11, 95% CI: 1.03 to 1.18), and the association between the two factors was linear in participants without coronary heart disease [117]. A study conducted at Soochow University in China found similar results [118]. In addition, TMAO was shown to increase the risk of stroke in the post-carotid artery stenting population [119].

In summary, despite the inconsistent results from previous studies, there is increasing evidence of the important role of TMAO in increasing the ischemic stroke risk. The mechanism underlying the TMAO-induced increased risk of ischemic stroke involves not only traditional risk factors but also key pathophysiological mechanisms that are still unclear but are worthy of focus and exploration in future studies.

## 5. TMAO and the Severity of Ischemic Stroke

TMAO not only increases the risk of ischemic stroke, it may also aggravate the severity of the condition. Wu et al. found higher plasma TMAO concentrations in patients with high NIHSS scores, and TMAO levels were positively correlated with initial NIHSS scores (*ρ* = 0.557; *p* < 0.001) and the cerebral infarct size on an MRI (*ρ* = 0.558; *p* < 0.001). In addition, an increase in the NIHSS score by 1.25 points (95% CI 1.12 to 1.38; *p* < 0.001) or the infarct volume by 1.62 mL (95% CI 1.27 to 1.97; *p* < 0.001) for every 1 μmol/L increase in plasma TMAO concentrations was found [120]. Sun et al. and Li et al. found similar results [53,110]. In diabetic patients with acute ischemic stroke, TMAO not only predicted the disease severity but also was correlated with the glycemic variability, which may promote early neurological deterioration [53]. Another study showed that, in patients experiencing their first ischemic stroke, elevated TMAO was associated with early neurological deterioration, which is defined as an increase in NIHSS scores by 2 or more points within 3 days [121]. Importantly, preclinical studies have shown that the gut microbiota impact the severity and prognosis of ischemic stroke via the TMAO pathway. In multiple murine stroke models, dietary supplementation with TMAO or choline has resulted in larger cerebral infarct sizes and lower motor function scores. In microbial community transplantation experiments, the direct contribution of the TMA/TMAO pathway to stroke severity was proven via knockout microbial cutC enzyme genes [122]. Post-stroke inflammatory cascades are associated with the ischemic stroke severity and involve the activation of multiple immune cells and the release of inflammatory cytokines and chemokines. Increased gut permeability and breakdown of the blood–brain barrier after a stroke allow the microbiota-derived metabolite TMAO to infiltrate the brain, and TMAO may lead to increased neurological impairment by further aggravating neuroinflammation. The NLRP3 inflammasome plays an important role in the inflammatory response after ischemic stroke. TMAO was found to activate the NLRP3 inflammasome, promoting the release of pro-inflammatory cytokines such as interleukin (IL)-1β. In addition, TMAO may increase the inflammatory response by activating microglia and astrocytes, upregulating the expression of P38, MAPK, and IL-1β [123]. Overall, the pathophysiological mechanism by which TMAO aggravates the severity of ischemic stroke remains unclear but may involve inflammation. Future research should focus on the above mechanisms and develop therapeutic strategies to reduce the severity and mortality associated with ischemic stroke to improve disease outcomes.

TMAO may also play a role in the occurrence and development of cerebral small vessel disease. Recent studies have shown that elevated levels of TMAO are associated with the periventricular white matter burden but not deep white matter hyperintensities (WMH), lacunes, and cerebral microbleeds [124]. Naruchorn et al. found an association between plasma TMAO levels and the WMH volume and acute lacunar infarction after adjustment for traditional vascular risk factors such as age, sex, hypertension, diabetes, and smoking status [125]. The mechanism underlying the TMAO-induced increases in WMH and the lacunes burden is not known, but it may be related to TMAO-induced endothelial dysfunction, which is a common pathophysiology in cerebral small vessel disease.

## 6. TMAO and the Prognosis and Recurrence of Ischemic Stroke

Ischemic stroke is associated with high disability, mortality, and recurrence rates, seriously threatening the health and life quality of patients. Recent studies have shown that elevated TMAO levels are associated with adverse functional outcomes following a stroke. After adjustment for traditional vascular risk factors, elevated TMAO levels were found to predict worse three-month functional outcomes (quartiles 4 vs. 1, odds ratio (OR) 5.65, 95% CI:2.87 to 13.45) and higher mortality risks (quartiles 4 vs. 1, OR 5.84, 95% CI: 3.05 to 16.12) in acute ischemic stroke patients [126]. Similar results were found in another study that used the modified Rankin scale (mRS) to evaluate functional outcomes at 3 months after stroke [127]. A study with an average follow-up period of 1.9 years found that after adjusting for age, diabetes, NIHSS scores, and hemoglobin levels, the TMAO concentration was predictive of major adverse cardiovascular events (HR 1.690, 95% CI: 1.030 to 2.771), including recurrent stroke, myocardial infarction, and death [128]. In addition, baseline TMAO levels could be used to predict major ischemic activities, including acute cerebral infarction, myocardial infarction, or death due to ischemic vascular events and adverse functional outcomes defined as mRS scores of ≥3 at 1 year following stroke [129]. However, Chen et al. found that TMAO was not associated with functional outcomes measured by the mRS score at 3 months following stroke in large-artery atherosclerotic stroke patients [130]. Another study that focused on the relationship between TMAO and post-stroke cognitive impairment found that TMAO was negatively associated with Mini-Mental State Examination (MMSE) scores but not MOCA scores. Li et al. also showed that TMAO plays a role in aging and age-related cognitive impairments [131]. The above research results suggest that elevated TMAO levels are associated with worse short–medium-term motor function outcomes as well as worse nonmotor outcomes such as cognitive dysfunction in ischemic stroke [132].

There is increasing evidence to suggest that TMAO increases the risk of stroke recurrence in ischemic stroke patients. Chen et al. showed, for the first time, that TMAO is independently associated with the recurrence of adverse vascular events, including transient ischemic attacks, ischemic stroke, and myocardial infarction (adjusted HR, 3.128; 95% CI, 1.018–9.610) at 3 months after large-artery atherosclerotic stroke [132]. The association between TMAO and the risk of stroke recurrence was not consistent among different stroke subtypes. TMAO was associated with stroke recurrence in the small-artery occlusion subtype (TOAST criteria) but not in others, such as large-artery atherosclerosis and cardioembolism [133]. Importantly, TMAO still significantly increased the risk of stroke recurrence in patients who received dual-antiplatelet and intensive lipid-lowering therapy [134]. This suggests that the potential mechanisms by which TMAO promotes the occurrence and development of ischemic stroke may involve not only high platelet reactivity and atherosclerosis but also other important pathophysiological mechanisms such as endothelial tissue factor expression, extrinsic clotting cascade activation, and others, which needed to be further investigated. In addition, besides the traditional treatment for the secondary prevention of ischemic stroke, targeted TMAO pathway therapy may also reduce the risk of stroke recurrence.

Despite the exciting findings from different studies, previous studies have been characterized by short follow-up periods, small sample sizes, and single methods of assessing functional outcomes, as well as a lack of subgroup analysis. Future cohort studies with large sample sizes should extend the follow-up period, perform a subgroup analysis, and use multi-dimensional and more detailed evaluation methods, such as functional magnetic resonance imaging, vascular ultrasound, computed tomography angiography (CTA), cognitive scale, and depression scales.

The mechanism by which TMAO impacts the prognosis and recurrence of ischemic stroke remains unclear. In two independent cohort studies, Arash et al. found that TMAO predicted the risk of incidental cardiovascular events (CVEs) (including stroke) in acute stroke patients, and this was associated with the pro-inflammatory monocyte concentration. Plasma TMAO levels were positively correlated with the intermediate CD14++CD16+ monocyte percentage [135]. This factor is pro-inflammatory, aggravates brain damage, and affects neurological recovery in the acute and subacute ischemic stroke phases [136]. In preclinical studies, the increase in TMAO induced by dietary choline supplementation was also associated with the pro-inflammatory monocyte subtype [137]. In brief, TMAO increased the CVE risk (including strokes) and impacted the prognosis, possibly by activating pro-inflammatory monocytes in ischemic stroke patients. Su et al. found that, after acute ischemic stroke, TMAO impaired neurological function recovery by inducing reactive astrocyte proliferation and glial scar formation via the Smad ubiquitination-related factor 2 (Smurf2)/activin-like kinase 5 (ALK5) signaling pathway [137]. These results suggest that TMAO may worsen ischemic stroke prognosis by affecting astrocyte function. In addition, alterations in the gut microbiota associated with TMAO synthesis increased the risk of stroke-associated infections, which are important factors in the prognosis of stroke patients. Whether TMAO affects ischemic stroke outcomes by increasing the infection risk needs to be further investigated. Of course, the mechanisms by which TMAO impacts the recurrence of ischemic stroke may also involve endothelial dysfunction, atherosclerosis, platelet hyper-reactivity, and thrombosis, as described above.

## 7. TMAO and Interventions

**Figure 3:**
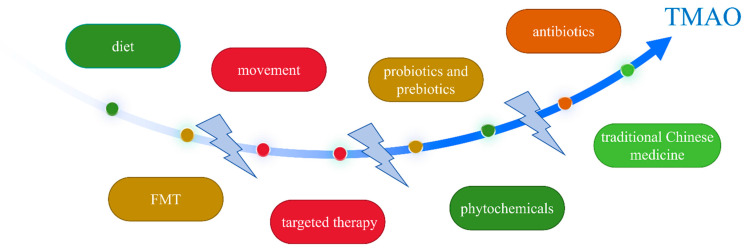
TMAO and interventions. The therapeutic strategies targeting gut dysbiosis and TMAO include diet, movement, probiotics and prebiotics, antibiotics, FMT, TMA- and TMAO-related targeted therapies, phytochemicals, and traditional Chinese medicine. FMT: fecal microbiota transplantation, TMA: trimethylamine, TMAO: trimethylamine-N-oxide.

Preclinical and clinical studies have proven that gut-microbiome-driven metabolite TMAO is an important ischemic stroke risk factor associated with the occurrence, severity, prognostic outcome, and recurrence of this condition. Therefore, therapeutic strategies targeting gut dysbiosis and TMAO have been the focus of recent research. Such strategies include diet, movement, probiotics and prebiotics, antibiotics, fecal microbiota transplantation (FMT), TMA- and TMAO-related targeted therapy, phytochemicals, and traditional Chinese medicine (Figure 3). We summarized and evaluated recent intervention strategies to provide evidence for future research targeting the gut dysbiosis–TMA–TMAO pathway for the prevention and treatment of ischemic stroke.

### 7.1. Diet

As mentioned above, diet choice is an important factor that affects the TMAO level. Diet is a key source of TMAO synthesis in the body and affects the composition, abundance, and function of the gut microbiota, which are related to key TMAO synthesis enzymes. The Mediterranean diet, which originates from the traditional cultures associated with the countries and regions along the Mediterranean coast, is a nutritionally recommended dietary pattern. It is based on plants supplemented with meat and is rich in fruits, vegetables, whole grains, legumes, fish, and olive oil. Previous studies have shown that the Mediterranean diet can reduce the risk of heart disease and stroke. Recent clinical studies have found that the Mediterranean and vegetarian or vegan diets are associated with lower TMAO levels [138], which may reduce the risk of developing cardiovascular and cerebrovascular diseases by reducing TMAO synthesis [139]. Interestingly, Melita et al. found that a short-term fasting-mimicking diet (FMD) that was characterized by the restriction of calories and animal proteins reduced TMAO synthesis, led to weight loss, and improved insulin sensitivity [140]. However, this conclusion still needs to be further verified by large-sample studies. In addition, a chronic red-meat-rich diet is not recommended because it increased TMAO levels by increasing TMAO production and reducing renal TMAO excretion in healthy volunteers [34]. It may be beneficial to reduce the intake of fat, as this increases circulating TMAO levels by impairing intestinal cell function [79]. A high-salt diet has been associated with several diseases such as hypertension and the promotion of TMAO production by inducing gut dysbiosis in rats [141]. Although seafood and fish are rich in TMAO, dietary fish sticks, cod, and salmon, but not shrimp or tuna, were found to cause a temporary increase in plasma TMAO levels [142]. In clinical studies, compared with non-fermented dairy products, fermented dairy product consumption resulted in lower postprandial circulating TMAO levels [143]. In preclinical studies, dietary fish oil supplements inhibited TMAO-induced atherogenesis more effectively than flaxseed oil [144]. In brief, dietary choices affect the cardiovascular and cerebrovascular risk by altering the gut microbiota composition and TMAO level. Currently, the Mediterranean diet is recommended for individuals at high risk of ischemic stroke, especially those with high circulating TMAO levels.

### 7.2. Exercise

Exercise is associated with the diversity and metabolic function of gut microbiota. Recent studies found that physical activity may affect gut-microbiota-derived metabolite TMAO production. Stavroula et al. found that moderate-to-vigorous physical activity, but not light physical activity, is associated with lower plasma TMAO concentrations, suggesting another possible mechanism by which physical exercise reduces cardiovascular and cerebrovascular disease risk [145]. Lifestyle intervention with a calorie-restricted diet combined with high-intensity physical exercise also appears to reduce TMAO production [146]. Additionally, exercise (voluntary wheel running) was shown to reduce TMAO levels and reverse TMAO-induced cognitive impairment in mice [147]. However, Daniel et al. found that interval training (1 h/day, 3 min at 90% and 50% heart rate peak) did not reduce TMAO levels [81]. Previous research findings suggest that exercise mode and intensity could be the key factors influencing the TMAO level. The underlying mechanism by which exercise affects TMAO levels may involve gut microbiota homeostasis and metabolic function [148], but this needs to be explored in future research.

### 7.3. Probiotics

Probiotics are active micro-organisms that are beneficial to human health. Supplementation with probiotics can help to maintain the gut microbiota balance, promote nutrient digestion and absorption, modulate the immune system, inhibit intestinal inflammation responses, and maintain oxide homeostasis and barrier integrity. Preclinical and clinical studies have found that supplementation with specific probiotic strains can effectively reduce TMAO levels. In healthy subjects, supplementation with *Bifidobacterium animalis subsp. lactis LKM512* reduced TMA levels and the abundance of microbiota associated with TMA synthesis in feces [149]. Wang et al. found that probiotics (*Bifidobacterium breve* and *Bifidobacterium longum*) reduced choline-diet-induced TMAO synthesis in mice by modulating the abundance of *Firmicutes*, such as *Ruminococcaceae UCG-009* and *Ruminococcaceae UCG-010* [150]. In coronary artery disease (CAD) patients, conventional drug treatment combined with *Bifidobacterium lactis Probio-M8* significantly improved clinical efficacy by modulating the gut microbiota and downregulating the TMAO level [151]. Liu et al. demonstrated that the gut *Lactobacillus rhamnosus GG* strain is responsible for mitigating the development of hypertension, a risk factor for stroke, by downregulating TMAO levels in rats [152]. A systematic review showed that *Lactobacillus rhamnosus GG* is the most efficient strain for reducing TMAO production. *Lactiplantibacillus plantarum LP1145*, *Lactobacillus plantarum ZDY04*, *Enterobacter aerogenes ZDY01*, and *Lactobacillus amylovorus LAM 1345* all effectively reduced TMAO levels in animals [153]. However, some studies have shown opposing results. Nabil et al. found that the consumption of 900 billion live bacteria/day did not alter circulating TMAO levels after exposure to a high-fat diet in healthy males. This intervention included a variety of strains, such as *Lactobacillus acidophilus DSM24735* and *Bifidobacterium longum DSM24736* [154]. Although circulating TMAO concentrations in people living with human immunodeficiency virus are related to subclinical atherosclerosis and higher vascular risk, a long-term high-dose probiotic intervention did not reduce TMAO levels [155]. In healthy men, similar results were found [156]. The beneficial effects of *Lactobacillus plantarum 299v* treatment on vascular endothelial function were shown to be independent of the TMAO concentration in male patients with stable CAD [157]. In summary, previous evidence suggests that only a few probiotic strains can reduce TMAO production. In addition, the clinical study results are inconsistent. More evidence for the effect of probiotics on TMAO levels and the related pathophysiological mechanisms is needed. Probiotics may be a promising treatment for the prevention and management of ischemic stroke in the future.

### 7.4. Prebiotics

Supplementation with prebiotics is another important intervention to maintain the intestinal microbiota balance and homeostasis. Maria et al. found that essential oils derived from thyme and oregano, which are commonly consumed in the Mediterranean diet, increase the relative abundance of the *Lactobacillus* genus and short-chain fatty acid levels and reduce TMAO levels and protein oxidation in the circulation, eventually improving cardiac metabolism and function in a murine model of cardiometabolic disorder [158]. Essential oil emulsions derived from parsley and rosemary reduced plasma TMAO and cytokine levels and increased thrombomodulin levels by increasing the concentration of beneficial gut commensal bacteria, reducing the ischemic heart disease risk [159]. However, inulin, an important prebiotic, may not be associated with TMAO production. Two recent clinical studies have found consistent results. Although the consumption of inulin-type fructans (10 g/day) improved the gut microbiome composition, TMA-producing gene clusters and plasma TMAO levels were not significantly affected in continuous peritoneal dialysis patients [160]. In addition, inulin may not be an effective intervention to reduce circulating TMAO levels, as demonstrated in participants at risk for type 2 diabetes [161]. There is currently less evidence to support the idea that prebiotic interventions can reduce the ischemic stroke risk by effectively reducing TMAO levels, so investigations are still needed.

### 7.5. Antibiotics

Antibiotic interventions affect the composition, abundance, and function of the gut microbiota. Reduced circulating TMAO concentrations caused by antibiotic interventions have been demonstrated in preclinical and clinical studies [25,103], suggesting that the gut microbiota play a key role in TMAO production. Recent studies have shown that antibiotic-induced intestinal microbiota depletion significantly reduces TMAO production, which is associated with the transition of acute kidney injury to chronic kidney disease [162]. However, the TMAO-inhibitory effect of broad-spectrum antibiotics disappears after drug withdrawal. Long-term antibiotic interventions can have a variety of adverse consequences, such as microbial imbalance in the body, liver and kidney dysfunction, antibiotic resistance, and increased susceptibility to infections. The balance between benefits and risks is the key to clinical intervention selection.

### 7.6. Fecal Microbiota Transplantation (FMT)

In recent years, FMT has been widely used in human and animal studies and has gradually become a promising method for the correction of gut microbial disorders and related diseases. Tousif et al. stated that FMT protects against ischemic injury by inhibiting neuroinflammation in ischemic stroke patients by upregulating the expression of Bcl-2 and reducing inflammatory factors (IL-17, IFN-γ, Bcl-2-associated X protein (Bax)) [163]. However, although vegan fecal microbiota transplantation was shown to affect gut microbiota composition, it was not associated with TMAO production in patients with metabolic syndrome [164]. Currently, there is a lack of scientific data to support the hypothesis that FMT reduces the risk of ischemic stroke by inhibiting TMAO production, and further studies are needed.

### 7.7. TMA- and TMAO-Related Targeted Therapies 

The synthesis of TMA (a precursor of TMAO) and TMAO is regulated by key gut microbiota enzymes such as cutC/D, cntA/B and yeaW/X, and the clinical intervention selected can significantly reduce TMA and TMAO levels. In addition, targeted FMO inhibition (key hepatic enzyme that oxidizes TMA to TMAO) can also reduce TMAO levels. Four main enzymes are involved in the production of TMA: cutC/D, cntA/B, betaine reductase, and TMAO reductase. In addition, the cntA/B homologous enzyme yeaW/X system can generate TMA from carnitine, choline, gamma-butylbetaine, and betaine. Specific gut microbiota choline utilization gene clusters encode catalytic and regulatory peptides, choline TMA-lyase (cutC), and choline TMA-lyase activator (cutD). Compared with *cutC*-containing bacteria, transplanting *cutC*-KO human commensal bacteria into germ-free mice significantly reduced the cerebral infarction size and neurological impairment after acute ischemic stroke by inhibiting choline-diet-induced TMAO levels [122]. This suggests that the targeted inhibition of cutC is a promising intervention that can inhibit the TMAO pathway [165,166]. The choline analog 3,3-dimethyl-1-butanol (DMB) is a competitive inhibitor of cutC and has been shown in multiple studies to reduce TMAO production. DMB alleviated atherosclerosis from intermittent hypoxia and hypercapnia by reducing TMAO production [167]. In mice, DMB prevented age-related endothelial dysfunction by reducing TMAO levels [168]. DMB alleviated TMAO-induced carotid remodeling [101] and age-related sympathetic excitation aggravated by TMAO [169]. Importantly, DMB, a natural product mainly found in red wine and olive oil, not only attenuated choline-diet-related atherosclerosis by reducing TMAO production, but it also did not seem to have any adverse effects on liver or kidney function, suggesting that it may be an effective and safe treatment that can reduce the ischemic stroke risk. In addition, fluoromethylcholine (FMC), a recently discovered novel cutC/D inhibitor, is effective, time dependent, irreversible, and does not affect commensal viability while reducing TMAO production. Oral FMC treatment was shown to mitigate diet-induced enhanced platelet hyper-reactivity and thrombosis by reducing TMA and TMAO production without causing a significant bleeding risk or toxic effects [170]. Since FMC was developed, several studies have demonstrated its effectiveness and safety in relation to reducing TMAO levels. The TMAO-induced upregulation of vascular endothelial tissue factor and vascular cell adhesion molecule, which contribute to the formation of arterial thrombosis, can be specifically blocked by FMC [108]. An intervention with FMC attenuated the choline-related initiation and progression of aneurysms by inhibiting TMAO production [171]. FMC also significantly reduced circulating TMAO concentrations by inhibiting cutC in tumor and diet-induced obesity mouse models [76,172]. Another choline structural analog, betaine aldehyde, was used to target the gut microbial enzyme choline TMA-lyase (cutC), which mildly to moderately inhibited cutC but only slightly reduced TMA production in whole-cell assays [173]. A metabolically stable peptidomimetic, Compound 5, has been shown to possess universal CutC-inhibitory activity in a variety of bacterial strains, unlike other targeted cutC inhibitors [174]. Interestingly, Lee et al. found that 3,6,7,8,2’,5’-hexamethoxyflavone reduced TMAO formation by significantly inhibiting cutC/D and downregulating FMO3 expression [175], which inhibited TMAO production by dual targeting, similar to berberine. In the gut, carnitine is oxidized by CntA/B, and its C–N bond is cleaved to produce TMA and malate hemialdehyde. Nobiletin and feruloylputrescine extracted from citrus peel have also recently been found to reduce TMA and TMAO production by targeting cntA/B enzymes [175,176]. Previous studies have confirmed that FMO3 knockout can significantly reduce gut-microbiota-dependent TMAO-induced atherosclerosis and thrombosis, suggesting that therapies targeting FMO3 may reduce the ischemic stroke risk [60,177,178]. 3,6,7,8,2’,5’-Hexamethoxyflavone and berberine may reduce TMAO levels by affecting FMO3 expression and function.

In conclusion, nonlethal interventions targeting gut microbial enzymes that limit systemic exposure of the host to the inhibitor may reduce the risk of TMAO-associated ischemic stroke. Previous studies have mainly focused on animal interventions and have not been applied to clinical practice, and relevant conclusions still need to be confirmed.

### 7.8. Phytochemicals, Traditional Chinese Medicines, and Others

There is mounting evidence to support the important role and potential of phytochemicals in regulating gut microbial homeostasis and TMAO production. Li et al. showed that puerarin, a natural compound, alleviates atherosclerosis by decreasing the abundance of *Prevotella copri*, which produces TMA, and inhibiting TMAO production in animal and human studies [179]. Geraniin has also been shown to alleviate atherosclerosis by inhibiting gut-microbiota-derived TMAO and reducing inflammatory responses in mice [96]. Additionally, other phytochemicals such as curcumin, allicin, taurine, protocatechuic acid, quinic acid, resveratrol, gypenosides, polyphenols from hickory nut, hawthorn fruit extract, mangiferin, ginger essential oil, and citral are involved in the correction of the gut microbiota imbalance and the reduction in TMAO levels, thus inhibiting atherosclerosis development [180,181,182,183,184,185,186,187,188,189]. The pathogenic mechanisms underlying the amelioration of TMAO-aggravated atherosclerosis by phytochemicals may involve the reshaping of the gut microbiota, the regulation of lipid and bile acid metabolism and macrophage polarization, the mitigation of inflammation and oxidation, and the improvement of vascular endothelial dysfunction. Although previous study results have confirmed that phytochemicals can reduce the risk of atherosclerosis and ischemic stroke, research data on the adverse effects of phytochemical drugs are lacking. Therefore, before the widespread application of phytochemical interventions, further large-sample studies are required to verify effectiveness and assess the adverse effects of this treatment strategy.

Recently, traditional Chinese medicines have been shown to alleviate TMAO-induced atherosclerosis. Ma et al. found that berberine, an active compound extracted from the traditional Chinese medicinemedicine coptidis rhizoma, interrupts atherosclerotic plaque formation via a vitamin-like effect inhibiting TMAO production in animal and human studies [190]. Previous studies have found similar results [191]. Additionally, Li et al. showed that the Qing-Xue-Xiao-Zhi formula (a traditional Chinese medicine) alleviates atherosclerosis by promoting lipid efflux and inhibiting macrophage lipid accumulation and inflammatory response [192]. The results of previous studies suggest that ethnic medical interventions are also promising for the prevention of atherosclerosis and ischemic stroke.

Ranitidine and finasteride reduce atherosclerosis and renal damage by increasing the richness and diversity of gut microbiota and inhibiting the synthesis and release of TMAO in mice [193]. The exploration of new pharmacological effects and applications of conventional drugs is also of interest.

## 8. Problems and Challenges

Despite extensive evidence showing a close relationship between TMAO and ischemic stroke, there are still many questions that need to be addressed. In addition, with the application of gut microbiota transplantation, gene editing, and other technologies, the focus of the research field has moved from association to causation. The exact molecular mechanisms by which TMAO enhances platelet hyper-reactivity and promotes thrombosis are not yet known. To determine the mechanisms by which TMAO exacerbates ischemic stroke severity and affects neurological recovery, a detailed molecular exploration is needed. Does TMAO function as a protein allosteric modifier, or does it activate or inhibit signaling pathways through classical receptor–ligand interactions? Future studies need to minimize confounding factors, establish causality, and focus on the molecular mechanisms by which TMAO is involved in the occurrence and development of cardiovascular and cerebrovascular diseases.

## 9. Conclusions

The gut microbiota communicate bidirectionally with ischemic stroke through the brain–gut axis, which is mediated by the nervous and immune systems, gut microbial components, and microbiota-derived metabolites, such as short-chain fatty acids and TMAO. Previously collected data show that gut-microbiota-dependent TMAO plays an important role in ischemic stroke risk factors, disease risk and severity, prognostic outcomes, and recurrence. TMAO is associated with a variety of ischemic stroke risk factors, such as hypertension, diabetes, dyslipidemia, obesity, and atrial fibrillation, and may be involved in their development through several mechanisms, including oxidative stress, immunoinflammation, sympathetic nerve excitation, endoplasmic reticulum stress, circadian rhythm and metabolism changes, and mitochondrial dysfunction. Importantly, TMAO aggravates atherosclerosis, a key causative factor for stroke, by affecting cholesterol metabolism and promoting foam cell formation and endothelial dysfunction, the mechanisms underlying which may be involved in bile acid metabolism, inflammation, oxidative stress, and pyroptosis. In addition, TAMO enhances platelet hyper-reactivity, increases the expression and activity of vascular endothelial tissue factors, and promotes thrombosis. Although the mechanisms are unclear, previous studies have shown that TMAO affects ischemic stroke severity and neurological recovery and increases the risk of recurrence. In short, TMAO is involved in the overall development of ischemic stroke.

Therefore, gut-microbiota-dependent TMAO interventions represent a new hotspot for the control of ischemic stroke events. In this paper, we reviewed and evaluated recent research results and concluded that the Mediterranean diet, exercise, probiotics, prebiotics, antibiotics, fecal microbiota transplantation, TMA- and TMAO-related targeted therapies, phytochemicals, and traditional Chinese medicines may be potential candidates for reducing TMAO levels to protect against and treat ischemic stroke.

## Data Availability

Data sharing is not applicable to this article as no new data were created or analyzed in this study.
